# The Relative Risk and Incidence of Immune Checkpoint Inhibitors Related Pneumonitis in Patients With Advanced Cancer: A Meta-Analysis

**DOI:** 10.3389/fphar.2018.01430

**Published:** 2018-12-11

**Authors:** Ke Ma, Yali Lu, Shanshan Jiang, Jiangong Tang, Xin Li, Yuyang Zhang

**Affiliations:** Department of Pharmacology, School of Life Science and Biopharmaceutics, Shenyang Pharmaceutical University, Shenyang, China

**Keywords:** immune checkpoint inhibitors, immune-related adverse events, pneumonitis, anti-PD-1/PD-L1 antibodies, nivolumab, pembrolizumab, atezolizumab

## Abstract

**Background:** Recently, immune checkpoint inhibitors (ICIs) have been proved one of the most promising anti-cancer therapy, series clinical trials have confirmed their efficacy. But they are also associated with distinctive set of toxic effects, which are recognized as immune-related adverse events. Among those immune-related adverse events, pneumonitis is rare, but it is often clinically serious and potentially life-threatening. Although many clinical trial results of PD-1/PD-L1 inhibitors had been reported incidence of pneumonitis, the knowledge based on the individual cohort data from each clinical trial is limited. So we conducted a meta-analysis of trials of PD-1/PD-L1 inhibitors in patients with advanced cancer and compared relative risk and incidence among different tumor types and therapeutic regimens. Such an analysis may provide important knowledge of this rare but clinically significant and potentially serious immune-related adverse event.

**Methods:** Electronic databases were used to search eligible literatures, include randomized controlled trials (RCTs) comparing immune checkpoint inhibitors vs. standard therapies. All-grade (1–4) or high-grade (3–4) pneumonitis events were extracted. The summary relative risk, summary incidence, and 95% confidence intervals were calculated.

**Results:** The incidence of all-grade and high-grade pneumonitis in non-small cell lung cancer (NSCLC) was significantly higher compared with other tumor types, such as Melanoma, urothelial carcinoma (UC), head and neck squamous cell carcinoma (HNSCC) (3.1% vs. 2.0%; *p* = 0.02, 1.4% vs. 0.6%; *p* = 0.03). The risk of all-grade pneumonitis was obtained from all patients in both experimental arm and control arm. Treatment with immune checkpoint inhibitors targeting PD-1/PD-L1 did significantly increase the risk of all-grade and high-grade pneumonitis compared with controls (fixed effects, RR: 4.70; 95% CI: 2.81–7.85; *p* < 0.00001, RR: 3.33; 95% CI: 1.68–6.59; *p* = 0.0006).

**Conclusion:** The incidence of immune checkpoint inhibitors related pneumonitis was higher in NSCLC than other tumor types. Patients treated with immune checkpoint inhibitor in experiment arms are more likely to experience any grade pneumonitis than control arms. These findings suggest that clinician need to draw more attention on this rare but serious adverse event.

## Introduction

In recent years, immunotherapy has become the fourth treatment mode of antitumor treatment. Especially the research of ICIs has made a significant breakthrough. By blocking PD-1/PD-L1 pathway, ICIs can enhance the effect of antitumor immune response of T cell (Pardoll, 2012; Postow et al., 2015a). ICIs have shown a significant improvement in PFS and OS compared with standard therapies in series clinical trials that include different type of tumors. Recently FDA has approved three agent nivolumab, pembrolizumab (PD-1inhibitor), and atezolizumab (PD-L1 inhibitor) for treatment of different advanced solid tumors.

Although the remarkable efficacy of ICIs has been shown, they are also associated with distinctive set of toxic effects, which are recognized as immune-related adverse event, such as pruritus, rash, diarrhea, colitis, hypophysitis, thyroiditis, pancreatitis, nephritis, elevated liver function tests, and pneumonitis (Fujii et al., 2017; Kumar et al., 2017; Nagai and Muto, 2018). Among those immune-related adverse events, pneumonitis is rare, but it is often clinically serious and potentially life-threatening (Abdel-Rahman et al., 2017). By increasing the activity of the immune system, ICIs can potentially cause immune-related adverse events to various organ systems (Michot et al., 2016). Recent study suggests that renal cell carcinomas (RCC) patients are more likely to experience immune-related anemia from PD-1 inhibitors, because existing tumor burden in the kidney may impair the capability for renal elimination of metabolites from the blood, leading to the accumulation of toxic metabolites (Sui et al., 2018). So it is interesting to hypothesize that existing tumor burden in different organ system may relate to different incidence of immune-related adverse effect. Considered the severity of pneumonitis which is potentially life-threatening if not promptly recognized and adequately treated, we aim to access its incidence and relative risk across different tumor types. Understanding incidence and relative risk of pneumonitis may offer help for its early detection and appropriate management.

## Methods

### Literature Search and Study Selection

We retrieved original articles from PubMed, EMBASE, Medline, Cochrane Controlled Trials Register Databases. The deadline for publication was August 31, 2017. For the systematic literature search, we used Medical Subject Heading terms (MeSH), which include the following: “ICIs,” “immune checkpoint blockade,” “nivolumab,” “pembrolizumab,” “atezolizumab,” “PD-1 inhibitor,” “PD-L1 inhibitor,” “cancer,” “tumor,” “carcinoma,” “phase II,” and “phase III.” The inclusion criteria of clinical trials include the following: (a) human clinical trials published in English; (b) phase II and III RCTs in patients with advanced or refractory cancer; (c) participants treated with single-agent PD-1/PD-L1 inhibitor or standard therapies; and (d) reporting of pneumonitis for all-grade (1–4) or high-grade (3–4). On basis of the Cochrane Handbook for Systematic Reviews of Interventions (Higgins et al., 2011), two independent investigators evaluated the risk of bias for the included clinical trials. Evaluated components include: Random sequence generation, allocation concealment, blinding, incomplete outcome data, and other sources of bias.

### Data Extraction

Two authors reviewed and extracted eligible literature, the disagreements were resolved by discussion; a third reviewer adjudicated the controversial parts. Data extraction was conducted on basis of the Preferred Reporting Items for Systematic Review and Meta-Analysis statement (Moher et al., 2009). The data extracted for each article were: first author’s name, year of publication, trial phase, masking, number of patients available for analysis, type of treatments, type of tumors, and number of all-grade (1–4) and high-grade (3–4) pneumonitis events in both populations.

### Statistical Method

The main objective of this study was to access the incidence of pneumonitis in patients with PD-1/PD-L1 treatment across different tumor types and to compare relative risk (RR) of pneumonitis between PD-1/PD-L1 inhibitors and standard therapies. We calculated the incidence of pneumonitis from the data available in each study. Incidence rates for each study are displayed in forest plots with 95% CIs estimated using exact binomial methods. The overall estimate for each set of graphs was based on the mean weighted incidence from 1000 samples bootstrapped from the subset of studies. We also used generalized linear models with generalized estimating equations to assess significant predictors of all-grade and high-grade pneumonitis. Two independent variables were included in each model: tumor types and therapeutic agents. The relative risk (RR), corresponding 95% confidence intervals (CIs) were also calculated in patients treated with PD-1/ PD-L1 inhibitors compared with standard therapies. We used delta method to calculate the 95% CIs (Morris and Gardner, 1988). The statistical heterogeneity among the selected studies was verified through the Cochrane’s Q statistic and I2 statistic (Higgins and Thompson, 2002; Higgins et al., 2003). If no statistically significant heterogeneity (*p* > 0.05 or *I*^2^< 50%) was shown among the results of the included trails, the pooled estimate was calculated based on the fixed-effects model. If significant heterogeneity (*p* < 0.05 or *I*^2^ > 50%) was observed in the analysis, a random effects model was used for the meta-analysis. The statistical analyses were performed using the Review Manager (version 5.3, The Cochrane Collaboration, Oxford, United Kingdom) and Stata version 12.0 (StataCorp, College Station, TX). We also used Begg’s and Egger’s test to identify the potential publication bias with funnel plot (Begg and Mazumdar, 1994; Egger et al., 1997).

## Results

### Literature Search

**FIGURE 1:**
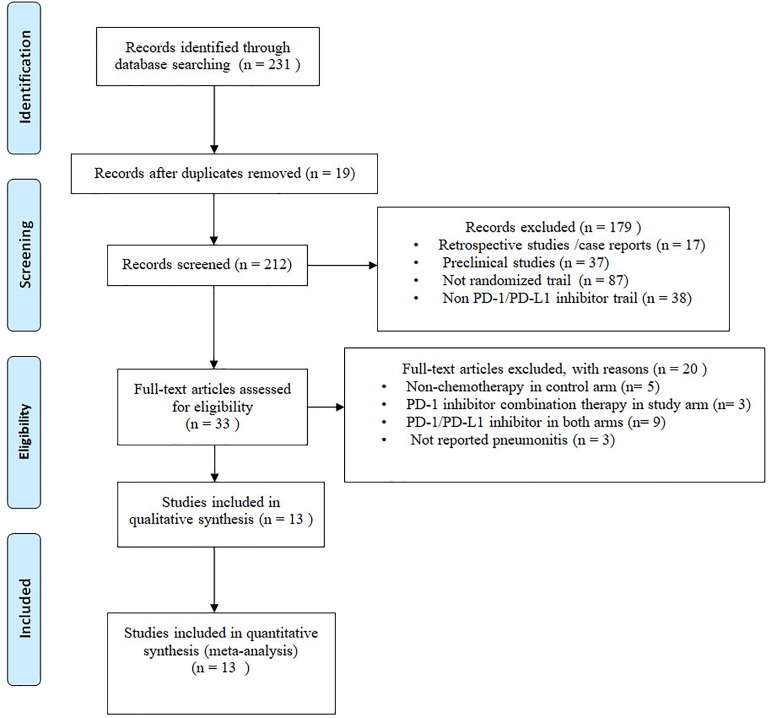
Flow diagram: selection process for the studies.

Two hundred thirty-one potential articles were retrieved from database searching, 19 studies were excluded due to duplicates. The remaining 212 articles were screened for titles and abstracts, on basis of our inclusion criteria, 179 articles were removed, 33 remaining articles were screened for full-text, and 20 articles were removed for different reasons. Eventually, we include 13 articles for final meta-analysis. The selection flow diagram is shown in (Figure 1).

### Study Characteristics

Final analysis include 13 random control trials (12 phase III and 1 phase II), comprising a total of 7092 patients (Supplementary Table S1) (Brahmer et al., 2015; Robert et al., 2015a,b; Borghaei et al., 2015; Ribas et al., 2015; Weber et al., 2015; Fehrenbacher et al., 2016; Ferris et al., 2016; Herbst et al., 2016; Reck et al., 2016; Bellmunt et al., 2017; Rittmeyer et al., 2017; Carbone et al., 2017). Among them, 4160 patients were assigned in the experimental arms with PD-1/PD-L1 inhibitor, while 2932 patients received standard therapies in the control arms.

### Incidence and Relative Risk of Pneumonitis

**FIGURE 2:**
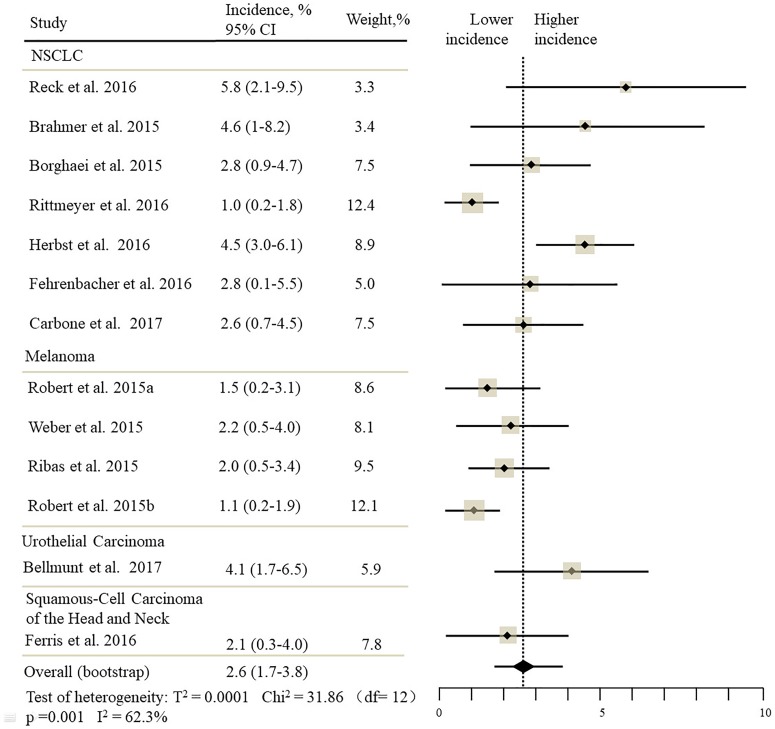
Incidence of all-grade pneumonitis during ICIs therapy in 13 studies in NSCLC, melanoma, urothelial carcinoma, head, and neck squamous cell carcinoma.

**FIGURE 3:**
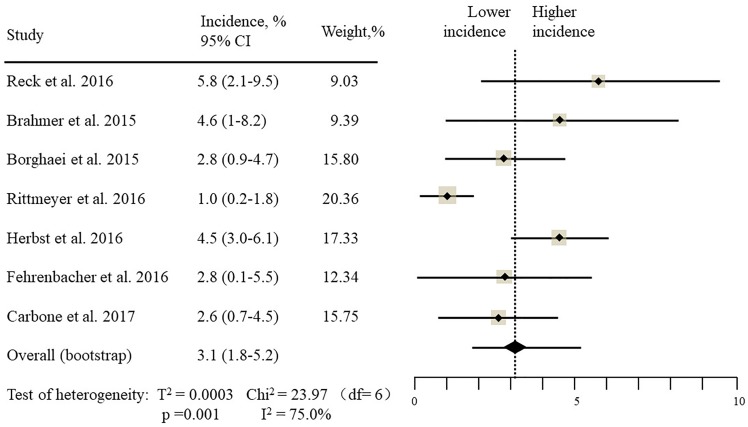
Incidence of all-grade pneumonitis during ICIs therapy in 7 NSCLC studies.

**FIGURE 4:**
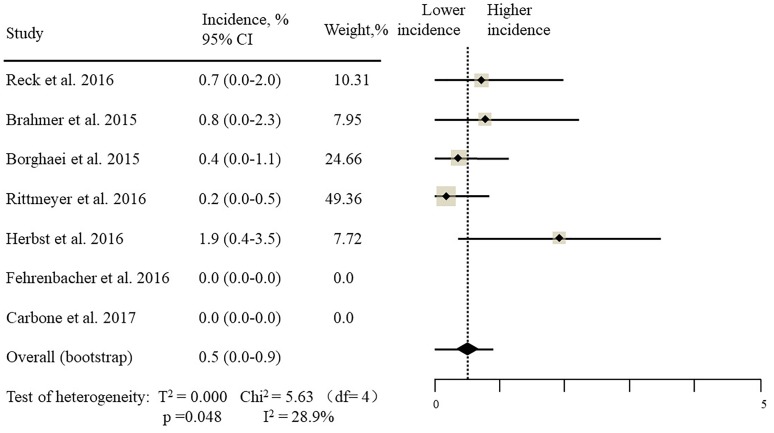
Incidence of all-grade pneumonitis in patients who did not undergo ICI therapy.

**FIGURE 5:**
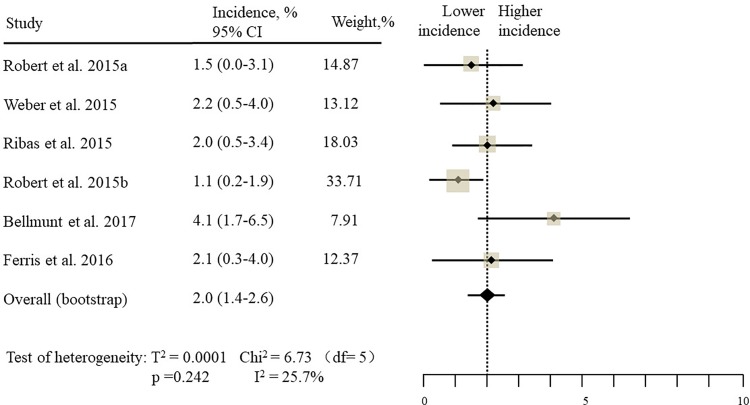
Incidence of all-grade pneumonitis during ICIs therapy in 6 other type tumor studies.

**FIGURE 6:**
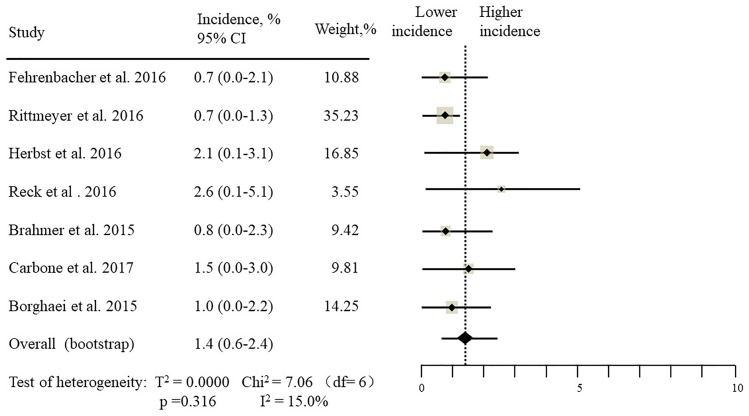
Incidence of high-grade pneumonitis during ICIs therapy in 7 NSCLC studies.

**FIGURE 7:**
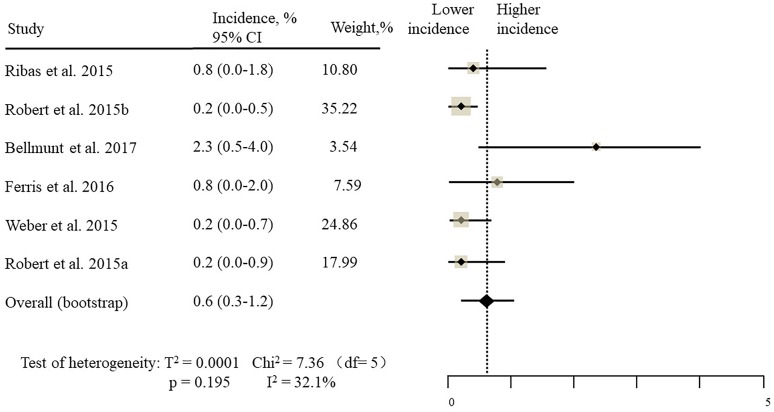
Incidence of high-grade pneumonitis during ICIs therapy in 6 other type tumor studies.

**FIGURE 8:**
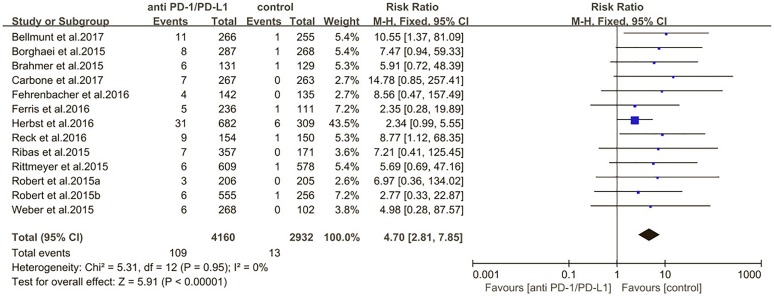
Relative risk for all-grade pneumonitis in patients treated with anti-PD-1/PD-L1 inhibitors or control.

**FIGURE 9:**
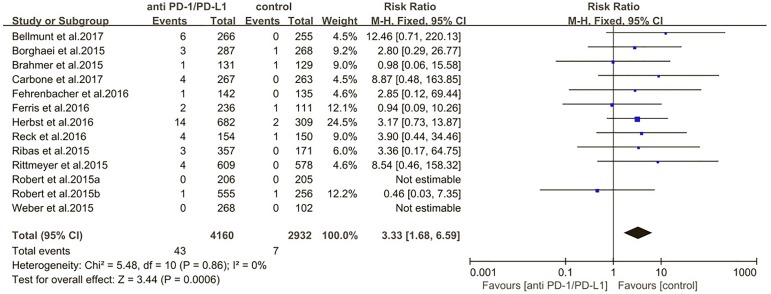
Relative risk for high-grade pneumonitis in patients treated with anti-PD-1/PD-L1 inhibitors or control.

The estimated incidences of pneumonitis were obtained from patients with melanoma, NSCLC, UC, and HNSCC in the experimental arms. The overall incidence of all-grade pneumonitis was 2.6% (95% CI, 1.7%–3.8%) (Figure 2 and Supplementary Table S2). Recent study suggests that NSCLC patients with ICIs therapy are more likely to experience immune-related pneumonitis than patients with chemotherapy (Khunger et al., 2017; Khoja et al., 2018; Luo et al., 2018). To substantiate the correlation between ICIs therapy and related pneumonitis in NSCLC patients, we conduct an analysis based on univariate generalized estimating equation models. We include ICIs as predictor, the incidence of all-grade pneumonitis in NSCLC patients treated with ICIs was significantly higher than in other patients who did not undergo ICIs in control arm (3.1% vs. 0.5%; *p* = 0.002) (Figures 3, 4). To compare the incidence of pneumonitis between NSCLC and other tumor types, we include tumor types alone as the predictor in the univariate generalized estimating equation models, the incidence of all-grade pneumonitis in NSCLC was significantly higher compared with other tumor types that include Melanoma, UC, and HNSCC (3.1% vs. 2.0%; *p* = 0.02) (Figures 3, 5), and the incidence of high grade pneumonitis in NSCLC was also higher compare with other tumor types (1.4% vs. 0.6%; *p* = 0.03) (Figures 6, 7). The risk of all-grade pneumonitis were obtained from all patients in both experimental arm and control arm, treatment with ICIs targeting PD-1/PD-L1 did significantly increase the risk of any grade pneumonitis compared with controls (fixed effects, RR: 4.70; 95% CI: 2.81–7.85; *p* < 0.00001). There was no heterogeneity (Chi^2^= 5.31; *p* = 0.95; *I*^2^ = 0%; Figure 8). Similarly, treatment with anti-PD-1/PD-L1 inhibitors did also significantly increase the risk of high-grade pneumonitis (fixed effect, RR: 3.33; 95% CI: 1.68–6.59; *p* = 0.0006). No heterogeneity was observed (Chi^2^ = 5.48; *p* = 0.86; *I*^2^ = 0%; Figure 9).

### Subgroups Analysis

**FIGURE 10:**
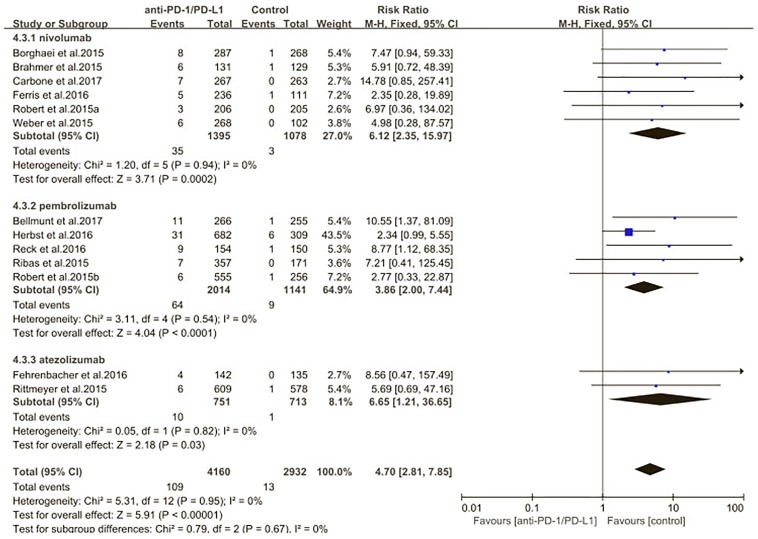
Relative risk of all-grade pneumonitis related to different type of immune checkpoint inhibitors (nivolumab, pembrolizumab, atezolizumab).

**FIGURE 11:**
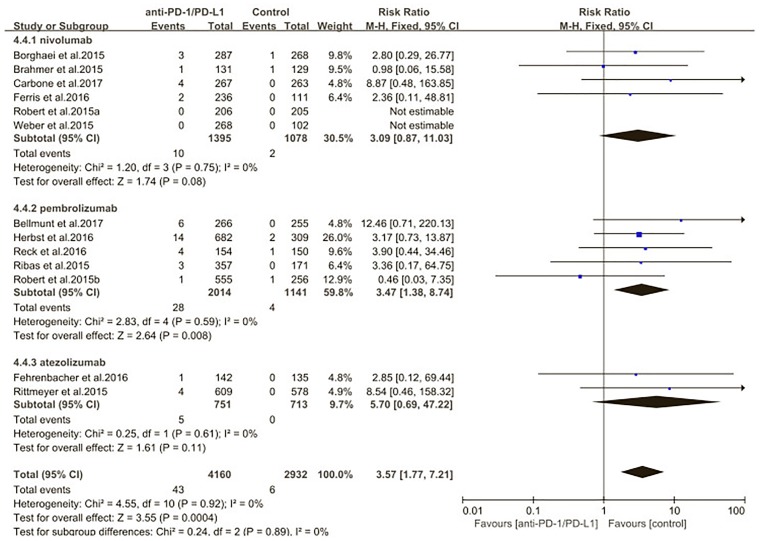
Relative risk of high-grade pneumonitis related to different type of immune checkpoint inhibitors (nivolumab, pembrolizumab, atezolizumab).

We also evaluated the risk of pneumonitis related to different type of ICIs (nivolumab pembrolizumab atezolizumab) (Figures 10, 11). Treatment with nivolumab did significantly increase the RR of all-grade pneumonitis compared with the control arm (RR: 6.12; 95% CI: 2.35–15.97; *p* = 0.0002) but did not significantly increase the RR of high-grade pneumonitis compared with the control arm (RR: 3.09; 95% CI: 0.87–11.03; *p* = 0.08). While treatment with pembrolizumab did significantly increase the RR of pneumonitis compared with the control arm, both in all-grade and in high-grade (RR: 3.86; 95% CI: 2.00–7.44; *p* < 0.0001; RR: 3.47; 95% CI: 1.38–8.74; *p* = 0.008). Treatment with atezolizumab did significantly increase the RR of all-grade pneumonitis compared with the control arm, but did not significantly increase the RR of high-grade pneumonitis compared with the control arm (RR: 6.65; 95% CI: 1.21–36.65; *p* = 0.03; RR: 5.7; 95% CI: 0.69–47.22; *p* = 0.11). However, no significant differences were found among nivolumab, pembrolizumab, and atezolizumab for both the all-grade (*p* = 0.89) and high-grade groups (*p* = 0.67) (Supplementary Table S3).

### Multivariable Analyses

**FIGURE 12:**
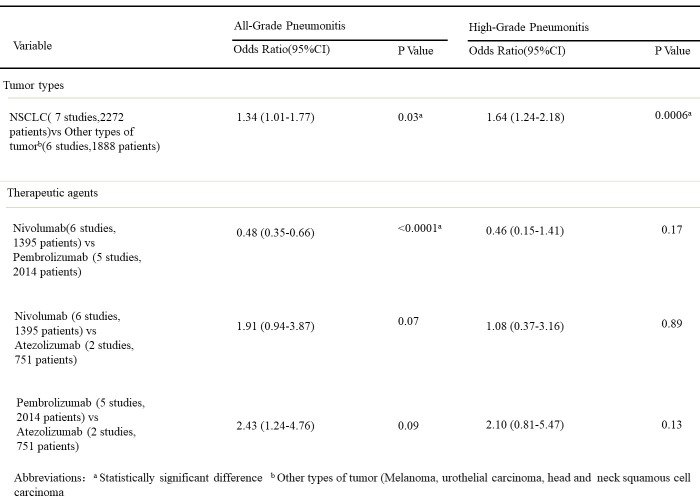
Multivariable analyses results for the incidence of PD-1/PD-L1 inhibitor-related pneumonitis.

The results of multivariable analyses are summarized for all-grade and high-grade pneumonitis (Figure 12). After adjusting for correlated incidence data and controlling for agents, patients with NSCLC were significantly more likely to experience all-grade pneumonitis(odds ratio [OR], 1.33; 95% CI, 1.01–1.76; *p* = 0.03) and high-grade pneumonitis (OR, 1.64; 95% CI, 1.23–2.18; *p* = 0.0006) compared with patients with other tumor types.

After adjusting for correlated incidence data and controlling for tumor types, we found significant differences between pembrolizumab and nivolumab for all grade pneumonitis, patients receiving pembrolizumab are more likely to experience pneumonitis than patients receiving nivolumab for all-grade pneumonitis (OR, 2.08; 95% CI, 1.52–2.85; *p* < 0.0001). However, the odds of experiencing high-grade pneumonitis were not significantly different between pembrolizumab and nicolumab. The odds of experiencing all-grade and high-grade pneumonitis were not significantly different between atezolizumab and nivolumab and between pembrolizumab and atezolizumab.

In the assessment of all-grade pneumonitis in the all ICIs therapy data, we found some study heterogeneity (*I*^2^ = 62.3%), which appeared to be concentrated in the studies of NSCLC (*I*^2^ = 75%), when we exclude the OAK study, the heterogeneity in studies of NSCLC significantly decreased (*I*^2^= 3%). In the OAK study, patients baseline characteristics include more EGFR KRAS mutation positive and ALK fusion positive patients, those patients who receive previous treatment with tyrosine kinase inhibitor are more likely to experience pneumonitis and lung inflammation (Choi et al., 2013; Awad and Nott, 2016; Chiang et al., 2016; Yoo et al., 2016). It seems that different patient inclusion criteria contributed to the observed heterogeneity. However, there was no evidence of publication bias for all-grade pneumonitis in the all ICIs therapy data (Begg test *p* = 0.2, Egger test *p* = 0.67) and in all studies of NSCLC data (Begg test *p* = 0.215, Egger test *p* = 0.13). Funnel plots for incidence of all-grade pneumonitis in all ICIs therapy studies and all studies of NSCLC are shown in Supplementary Figures S1, S2.

### Quality Assessment and Publication Bias

On basis of risk of bias graphs and risk of bias summaries, we used Review Manager 5.3 to evaluate the quality of each study from five parameters which include random sequence generation, allocation concealment, blinding, incomplete outcome data, and other sources of bias. Study that contains information of all parameters was evaluated as low risk. On the contrary, if there was no information at all, the study was evaluated as high risk. Study with partial or unclear information was evaluated as unclear risk. However, the overall risk of bias was evaluated as low risk and the quality of all studies was qualified (Supplementary Figures S3, S4). The funnel plots for relative risk of pneumonitis showed that no publication bias existed in our meta-analysis, each trails symmetrically distributed on both sides of the funnel (Supplementary Figures S5, S6).

## Discussion

In recent years, ICIs have become the most popular therapeutic regimen that concerns the treatment of various type of cancer. Although they have demonstrated promising prolonged PFS and OS associated with fewer adverse effects (AEs) in series type of cancer, immune-related adverse effects are completely different from traditional chemotherapy and targeted therapies related AEs. They have distinctive characteristics, including particular tendency for specific organs, no linear dose-dependent correlation, and potential late onset (Kadono, 2017). Among those immune-related adverse effects, pneumonitis is often clinically serious and potentially life-threatening. Therefore, it is important for us to deeply know immune-related pneumonitis for an adequate clinical management.

A higher incidence of pneumonitis among patients with NSCLC was observed in our study. There are following possible reasons: (1) extant tumor burden of primary lesion which localized in the lung may limits the lung tolerance to ectogenic stress and injury; (2) these patients are more likely to occur drug-related lung toxic effects because of tobacco use and underlying lung conditions, including pulmonary fibrosis and chronic obstructive pulmonary disease (Bouros et al., 2002; Toh et al., 2004).

The higher incidence of high-grade (3–4) pneumonitis among patients with NSCLC may also be explained by similar reasons. The incidence for all-grade and high-grade pneumonitis remained significantly higher for NSCLC compared with other type of tumors in the multivariable analyses after controlling for tumor types, adding more strength of evidence to this observation. Although none of the RCTs included in our study showed pneumonitis-related death, several phase I clinical trials of ICIs in patients with NSCLC have reported pneumonitis-related death, indicating that pneumonitis is an serious and potentially life-threatening adverse event. These results emphasize that clinicians should have necessary awareness on the possibility of pneumonitis and carefully monitor those NSCLC patients who treated with ICIs therapy.

The etiology of immune-related pneumonitis contributes to a potentially life-threatening toxicity related to PD-1/PD-L1 inhibitor. Two phase I clinical trials testing the safety of nivolumab reported pneumonitis-related death in one case(1.1%) (Postow et al., 2015b) and in three cases (2.3%) (Gettinger et al., 2015), respectively. Two clinical trials in phase I/III testing the safety of pembrolizumab also reported pneumonitis-related death in one case (0.2%) (Garon et al., 2015) and in three case (0.5%) (Herbst et al., 2016), respectively. According to previous research findings, immune related pneumonitis can independently develop during ICIs therapy without correlation of tumor type or dose level. As more anti PD-1/PD-L1 inhibitors are approved for clinical practice, physician need to draw more attention on this rare but serious adverse event. In pathological perspectives, drug induced a specific hyperactivation of T-cell immune response, which is responsible for cross-reactivity to normal tissue, causing the complication of autoimmune healthy tissue damage that clinically appears as immune related pneumonitis (Koelzer et al., 2017). In clinical practice, the manifestation of immune related pneumonitis includes rapidly exacerbated symptoms that develop to acute respiratory failure, solitary radiologic alterations with completely asymptomatic, mildly symptomatic cases characterized by dyspnea and dry cough (Nishino et al., 2015, 2016).

To accurately manage this rare but serious adverse effect, clinician should focus on its severity. For mild-to-moderate pneumonitis, which are commonly ephemeral, appropriate management include treatment discontinuation and observation. For serious pneumonitis, immunosuppressive therapy is fundamental. By counteracting the drug-mediated hyperactivated immune system which is responsible for auto-immune damage to healthy tissues, it can significantly relieve clinical symptoms. In some cases with severe symptoms, additional immunosuppressant may be required, such as infliximab (antitumor necrosis factor antibody) and mycophenolate mofetil (Friedman et al., 2016).

The current meta-analysis found that immune check point inhibitor significantly increase both incidence and risk of any-grade and high-grade pneumonitis compared with standard therapies. Although with remarkable efficacy and well safety, several ICIs, such as nivolumab, pembrolizumab, atezolizumab, Avelumab, and Durvalumab, have been approved by FDA for treatment of different type of cancer. However, the risk of developing an immune related pneumonitis in patients treated with these immune check point inhibitors is consistent and should always be taken into consideration by clinicians (Kazandjian et al., 2016; Antonia et al., 2017; Apolo et al., 2017; Chuk et al., 2017; Hazarika et al., 2017; Larkins et al., 2017; Ning et al., 2017; Powles et al., 2017; Weinstock et al., 2017; Patel et al., 2018).

## Limitation

Limitations of our analysis include inadequate inclusion of other immune checkpoint inhibitor and other tumor types. Our analysis focused on ICIs-related pneumonitis during RCTs of PD-1/PD-L1 inhibitors and did not include other ICI such as CTLA-4 inhibitor because of the paucity of published data at the time of data collection of this study. Similarly, our study focused on melanoma, NSCLC, UC, and HNSCC. Other tumor types, such as colorectal cancer or lymphoma, were not included because of the limited number of published reports with small sample sizes. Further studies are needed when more data become publicized to compare the incidence of pneumonitis among different agents targeting in the PD-1/PD-L1 pathway in a larger variety of tumors.

## Conclusion

Immune check point inhibitor-related pneumonitis represents a rare but often clinically serious and potentially life-threatening toxic effect. A comprehensive understanding of its clinical manifestation, continuous monitoring of symptoms, accurate diagnosis, and prompt immunosuppressive therapy are required to avoid potentially life-threatening progression. Because of a significant lack of knowledge of this adverse effect in terms of its risk factors, diagnostic strategy, and optimal treatment guidelines, large-scale systematic investigations are needed on the incidence and risk of pneumonitis across a larger variety of ICIs and tumor types. In the future, with more knowledge about immune-mediated pneumonitis, clinicians can overcome the previous concern to this life threatening adverse effect.

## Author Contributions

KM, YZ, and XL did the conception and design. YL and SJ did the collection and assembly of data. KM, YL, SJ, and JT did the analysis and interpretation. KM, YL, SJ, and JT wrote the manuscript. All authors did the final approval of the manuscript and were equally accountable for all aspects of the work.

## Conflict of Interest Statement

The authors declare that the research was conducted in the absence of any commercial or financial relationships that could be construed as a potential conflict of interest.

## Abbreviations

NSCLC: non-small-cell lung cancer
UC: urothelial carcinoma
HNSCC: head and neck squamous cell carcinoma
PD-1: programmed cell death protein 1
PD-L1: programmed death-ligand 1
ICIs: immune checkpoint inhibitors
