# Supplementary material for: The Relative Risk and Incidence of Immune Checkpoint Inhibitors Related Pneumonitis in Patients With Advanced Cancer: A Meta-Analysis
Source: Front Pharmacol. 2018 Dec 11;9:1430. doi: 10.3389/fphar.2018.01430 (PMC6297260; doi:10.3389/fphar.2018.01430)
Supplement: Supplementary file 1 [file Data_Sheet_1.docx]

**The relative risk and incidence of immune checkpoint inhibitors related pneumonitis in patients with advanced cancer: a meta-analysis**

Ke Ma, Yali Lu, Shanshan Jiang, Jiangong Tang, Xin Li, Yuyang Zhang*

Department of Pharmacology, School of Life Science and Biopharmaceutics, Shenyang Pharmaceutical University, Shenyang, China

***Correspondence**

Yuyang Zhang, PhD

E-mail: 13614053862@163.com

**Table S1 Selected clinical trials for meta-analysis**

| **Trial Name** | **First Author** | **Year** | **Trials Design** | | | | | | | | | **Patients characteristics** | | | **Study results** | | | **Jadad score** |
| --- | --- | --- | --- | --- | --- | --- | --- | --- | --- | --- | --- | --- | --- | --- | --- | --- | --- | --- |
|  |  |  | **Phase of study** | **N of arms** | **Experimental Arm** | | | | **Control arm** | | | **Type of cancer** | **Disease Stage** | **Line of therapy** | **Median Follow-up**  **(mos)** | **Median treatment duration (mos)**  **Exp./Ctr** | **mOS (mos)**  **Exp./Ctr** |  |
|  |  |  |  |  | Drug | Dosage | N Patients^*^ | Age | Drug | N Patients^*^ | Age |  |  |  |  |  |  |  |
| CHECKMATE 017 | Brahmer et al. | 2015 | 3 | 2 | NIVOLUMAB | 3 mg/Kg q14 | 131 | 62 | Docetaxel | 129 | 64 | NSCLC | IIIB or IV | 2^nd^ line, after one prior platinum-based chemotherapy | Minimum 11 | 8 doses/3 doses | 9.2 vs 6.0  HR 0.59;  (0.44-0.79; P<0.001) | 2 |
| CHECKMATE 057 | Borghaei et al. | 2015 | 3 | 2 | NIVOLUMAB | 3 mg/Kg q14 | 287 | 61 | Docetaxel | 268 | 64 | NSCLC | IIIB or IV | 2^nd^ line, after one prior platinum-based chemotherapy | Minimum 13.2 | 6 doses/4 doses | 12.2 vs 9.4  HR 0.73;  (0.59-0.89; P=0.002) | 2 |
| CHECKMATE 066 | Robert et al. | 2014 | 3 | 2 | NIVOLUMAB + Placebo | 3 mg/Kg q14  placebo q21 | 206 | 64 | Dacarbazine + Placebo | 205 | 66 | MELANOMA  BRAF WT | III or IV | 1^st^ line | 8.9 (nivolumab)  6.8(dacarbazine) | NR | Not reached vs 10.8  HR 0.42;  (0.25-0.73; P<0.001) | 5 |
| CHECKMATE 037 | Weber et al. | 2015 | 3 | 2 | NIVOLUMAB | 3 mg/Kg q14 | 268 | 59 | Chemotherapy | 102 | 62 | MELANOMA | IIIC or IV | 2^nd^ line, after ipilimumab, or Ipilimumab and BRAF-inhibitor | 8.4 | 5.3 / 2.0 | NR | 3 |
| CHECKMATE 141 | Ferris et al. | 2016 | 3 | 2 | NIVOLUMAB | 3 mg/Kg q14 | 236 | 59 | Cetuximab  or  Docetaxel  or  Methotrexate | 111 | 61 | SCCHN | Recurrent or IV | After one prior platinum-based chemotherapy | 5.1 | 1.9 / 1.9 | 7.5 vs 5.1  HR 0.70;  (0.51-0.96; P=0.010) | 3 |
| CHECKMATE 026 | Carbone et al | 2017 | 3 | 2 | NIVOLUMAB | 3 mg/Kg q14 | 271 | 63 | Chemotherapy | 270 | 65 | NSCLC | IIIB or IV | 1^st^ line | 13.5 | 3.7/3.4 | 14.4 vs 13.2  HR 1.02  (0.80-1.30 P=0.25) | 3 |
| KEYNOTE 024 | Reck et al. | 2015 | 3 | 2 | PEMBROLIZUMAB | 200 mg q 21 | 154 | 64.5 | Chemotherapy | 150 | 66 | NSCLC (PD-L1+) | IV | 1^st^ line | 11.2 | 7.0 / 3.5 | NR vs NR  HR 0.60  (0.41-0.89; P=0.005) | 3 |
| KEYNOTE 006 | Robert et al. | 2016 | 3 | 3 | PEMBROLIZUMAB | 10 mg/Kg q14 | 278 | 61 | Ipilimumab | 256 | 62 | MELANOMA | III or IV | ≤ 1 previous systemic therapy | 7.9 | 164 days / 151 days / 50 days | Not reached | 2 |
|  |  |  |  |  |  | 10 mg/Kg q21 | 277 | 63 |  |  |  |  |  |  |  |  | Not reached |  |
| KEYNOTE 002 | Ribas et al. | 2015 | 2 | 3 | PEMBROLIZUMAB | 2 mg/Kg q21 | 178 | 62 | Chemotherapy | 171 | 63 | MELANOMA | III or IV | 2^nd^ line or more | 10.0 | 113 days / 145 days / 61 days | NR | 3 |
|  |  |  |  |  |  | 10 mg/Kg q21 | 179 | 60 |  |  |  |  |  |  |  |  |  |  |
| KEYNOTE 010 | Herbst et al. | 2016 | 3 | 3 | PEMBROLIZUMAB | 2 mg/Kg q21 | 339 | 63 | Docetaxel | 309 | 62 | NSCLC (PD-L1+) | Advanced | 2^nd^ line, after one prior platinum-based chemotherapy | 13.1 | 3.5 / 3.5 / 2.0 | 10.4 vs 8.5  HR 0.71;  (0.58-0.88; P=0.0008) | 3 |
|  |  |  |  |  |  | 10 mg/Kg q21 | 343 | 63 |  |  |  |  |  |  |  |  | 12.7 vs 8.5  HR 0.61;  (0.49-0.75; P<0.0001) |  |
| KEYNOTE 045 | Bellmunt et al. | 2017 | 3 | 2 | PEMBROLIZUMAB | 200 mg q 21 | 270 | 67 | Chemotherapy | 272 | 65 | Urothelial Carcinoma | Advanced | 2^nd^ line, after one prior platinum-based chemotherapy | 14.1 | 3.5/1.5 | 8.0 vs5.2  HR 0.73  (0.59–0.91  P=0.002) | 3 |
| OAK | Rittmeyer et al | 2016 | 3 | 2 | ATEZOLIZUMAB | 1200mg q 21 | 425 | 63 | Docetaxel | 425 | 64 | NSCLC | IIIB or IV | 2^nd^ line, after one prior platinum-based chemotherapy | 21 | 3.4/2.1 | 13.8 vs 9.6  HR 0.73  (0.62-0.87 p=0.0003) | 3 |
| POPLAR | Fehrenbache et al | 2016 | 3 | 2 | ATEZOLIZUMAB | 1200mg q 21 | 144 | 62 | Docetaxel | 143 | 62 | NSCLC | IIIB or IV | 2^nd^ line, after one prior platinum-based chemotherapy | 14.8 | 3.7/2.1 | 12.6 vs 9.7  HR 0.73  (0.53-0.99 p=0.040) | 3 |

* Safety population, NR=not reported

**Table S2: Overall incidence of all- and high-grade** **pneumonitis from all included clinical trials.**

| **Study** | **Drug** | **Cancer Type** | **Evaluable Patients** | | **Pulmonary toxicity** | | | | | | | | |
| --- | --- | --- | --- | --- | --- | --- | --- | --- | --- | --- | --- | --- | --- |
|  |  |  |  |  | **Any-Grade** | | | | **High-Grade** | | | | |
|  |  |  | **Anti-PD-1/PD-L1** | **Control** | **Anti-PD-1/PD-L1** | | **Control** | | **Anti-PD-1/PD-L1** | | **Control** | | |
|  |  |  |  |  | **N**  **Events** | **Incidence** | **N Events** | **Incidence** | **N Events** | **Incidence** | **N Events** | **Incidence** |  |
| CHECKMATE 017 | NIVOLUMAB | NSCLC (squamous) | 131 | 129 | 6 | 4.6% | 1 | 0.8% | 1 | 0.8% | 1 | 0.8% |  |
| CHECKMATE 057 | NIVOLUMAB | NSCLC  (non-squamous) | 287 | 268 | 8 | 2.8% | 1 | 0.3% | 3 | 1.0% | 0 | 0 |  |
| CHECKMATE 066 | NIVOLUMAB | MELANOMA  BRAF WT | 206 | 205 | 3 | 1.5% | 0 | 0 | 0 | 0 | 0 | 0 |  |
| CHECKMATE 037 | NIVOLUMAB | MELANOMA | 268 | 102 | 6 | 2.2% | 0 | 0 | 0 | 0 | 0 | 0 |  |
| CHECKMATE 026 | NIVOLUMAB | NSCLC | 267 | 263 | 7 | 2.6% | 0 | 0 | 0 | 0 | 0 | 0 |  |
| CHECKMATE 141 | NIVOLUMAB | HNSCC | 236 | 111 | 5 | 2.1% | 1 | 0.9% | 2 | 0.8% | 0 | 0 |  |
| KEYNOTE 024 | PEMBROLIZUMAB | NSCLC (PD-L1+) | 154 | 150 | 9 | 5.8% | 1 | 0.7% | 4 | 2.6% | 1 | 0.7% |  |
| KEYNOTE 006 | PEMBROLIZUMAB | MELANOMA | 555 | 256 | 6 | 1.1% | 1 | 0.4% | 1 | 0.2% | 1 | 0.4% |  |
| KEYNOTE 002 | PEMBROLIZUMAB | MELANOMA | 357 | 171 | 7 | 2.0% | 0 | 0 | 3 | 0.8% | 0 | 0 |  |
| KEYNOTE 010 | PEMBROLIZUMAB | NSCLC (PD-L1+) | 682 | 309 | 31 | 4.5% | 6 | 1.9% | 14 | 2.1% | 2 | 0.6% |  |
| OAK | ATEZOLIZUMAB | NSCLC | 609 | 578 | 6 | 1.0% | 1 | 0.2% | 4 | 0.7% | 0 | 0 |  |
| POPLAR | ATEZOLIZUMAB | NSCLC | 142 | 135 | 4 | 2,8% | 0 | 0 | 1 | 0.7% | 0 | 0 |  |
| TOTAL EVENTS | | | 4160 | 2932 | 109 | 2.6% | 13 | 0.4% | 43 | 1.0% | 6 | 0.2% |  |

**Table S3:** **Sub-group analyses by type of therapy agent**

| **TYPE OF THERAPY** | **Study** | **N Patients** | | **N Events** | | | | **Incidence** | | | | **Risk Ratio** | |
| --- | --- | --- | --- | --- | --- | --- | --- | --- | --- | --- | --- | --- | --- |
|  |  |  |  | **Any-Grade** | | **High-Grade** | | **Any-Grade** | | **High-Grade** | | **Any-Grade** | **High-Grade** |
|  |  | **Anti-PD-1/PD-L1** | **Control** | **Anti-PD-1/PD-L1** | **Control** | **Anti-PD-1/PD-L1** | **Control** | **Anti-PD-1/PD-L1** | **Control** | **Anti-PD-1/PD-L1** | **Control** |  |  |
| NIVOLUMAB | CHECKMATE 017 | 1395 | 1078 | 35 | 3 | 10 | 2 | 2.5% | 0.3% | 0.7% | 0.2% | 6.12  [2.35 - 15.97]  P=0.0002 | 3.09  [0.87 - 11.03]  P=0.08 |
|  | CHECKMATE 037 |  |  |  |  |  |  |  |  |  |  |  |  |
|  | CHECKMATE 026 |  |  |  |  |  |  |  |  |  |  |  |  |
|  | CHECKMATE 057 |  |  |  |  |  |  |  |  |  |  |  |  |
|  | CHECKMATE 066  CHECKMATE 141 |  |  |  |  |  |  |  |  |  |  |  |  |
| PEMBROLIZUMAB | KEYNOTE 006 | 2014 | 1270 | 64 | 9 | 28 | 4 | 3.1% | 0.7% | 1.4% | 0.3% | 3.86  [2.00 - 7.44]  P<0.0001 | 3.47  [1.38 - 8.47]  P=0.008 |
|  | KEYNOTE 002  KEYNOTE 010  KEYNOTE 024  KEYNOTE 024 |  |  |  |  |  |  |  |  |  |  |  |  |
|  |  |  |  |  |  |  |  |  |  |  |  |  |  |
| ATEOLIZUMAB | OAK  POPULAR | 751 | 713 | 10 | 1 | 5 | 0 | 1.3% | 0.1% | 0.6% | 0 | 6.65  [1.21 - 36.35]  P=0.03 | 5.7  [0.69 - 47.22]  P=0.11 |
| TEST FOR SUBGROUP DIFFERENCES | | | | | | | | | | | | P=0.89 | P=0.76 |

**Figure S1. Funnel plots for incidence of all-grade pneumonitis in all ICIs therapy studies**

**
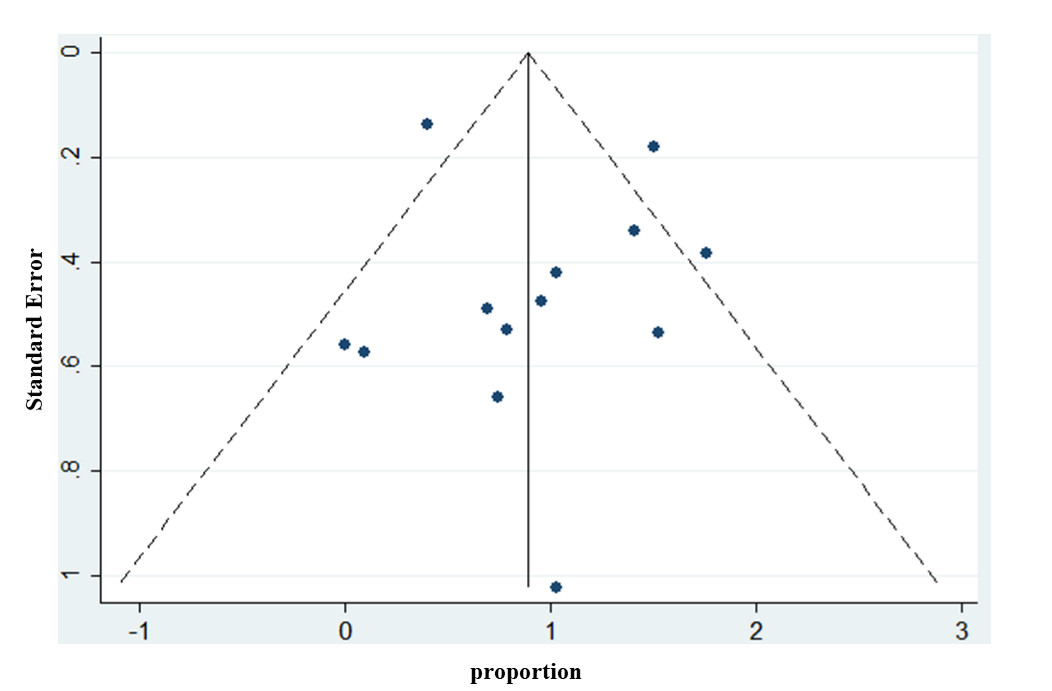
**

**Figure S2. Funnel plots for incidence of all-grade pneumonitis in all studies of NSCLC**

**
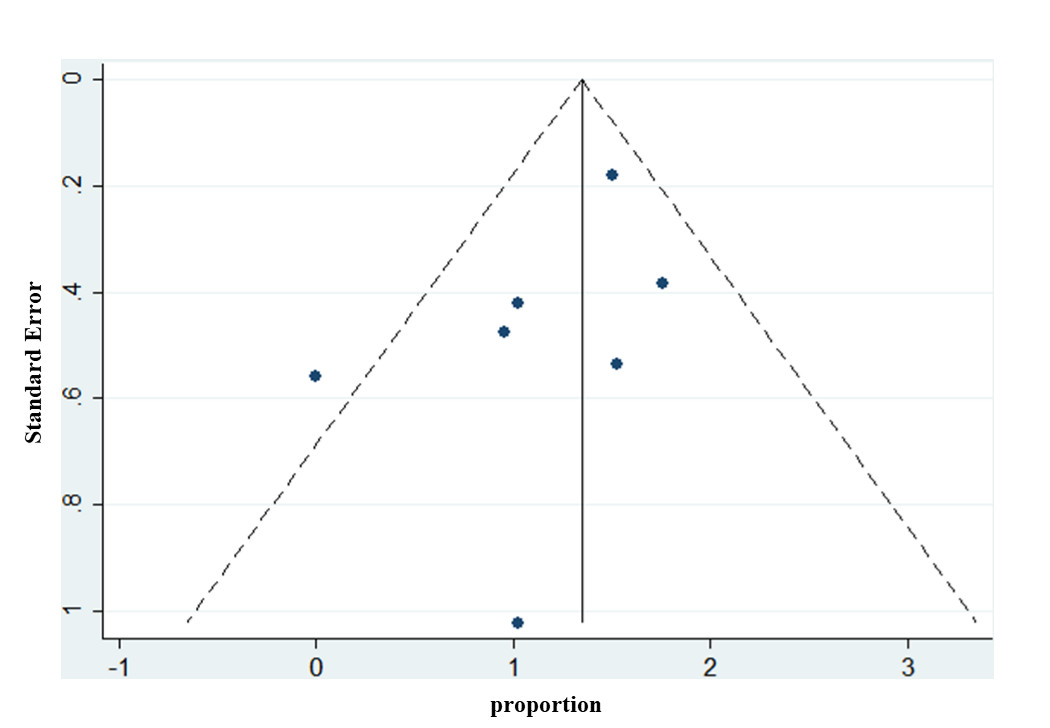
**

**Figure S3.** **Risk of bias graphs**

**
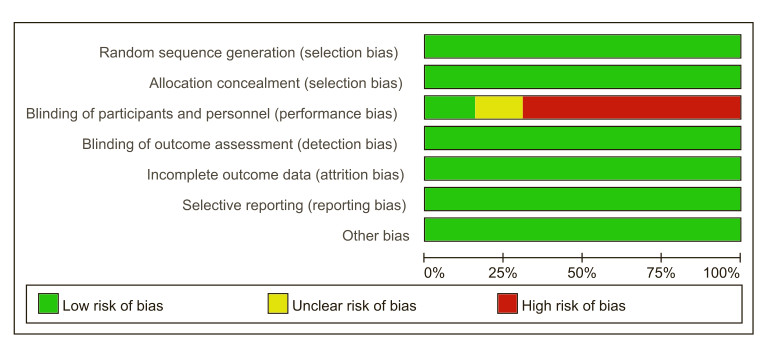
**

**Figure S4.** **Risk of bias graphs**

**
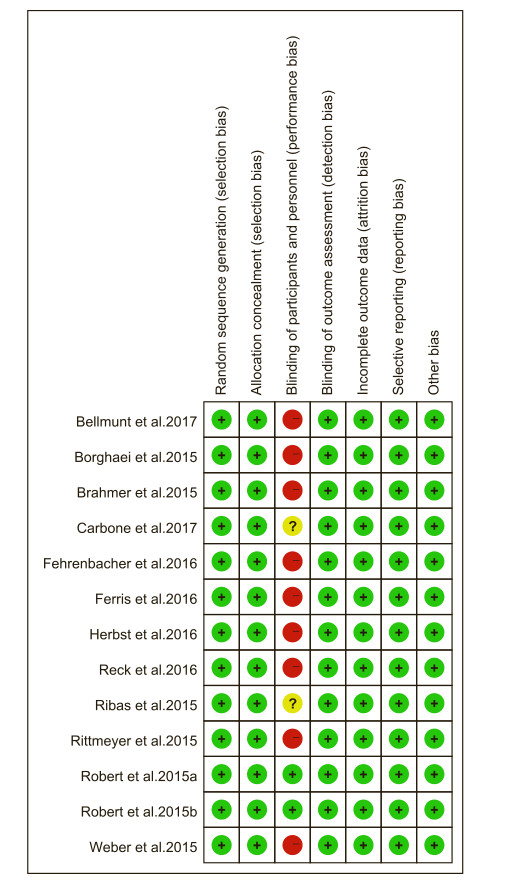
**

**Figure S5.** **The funnel plots for relative risk of all grade pneumonitis**

**
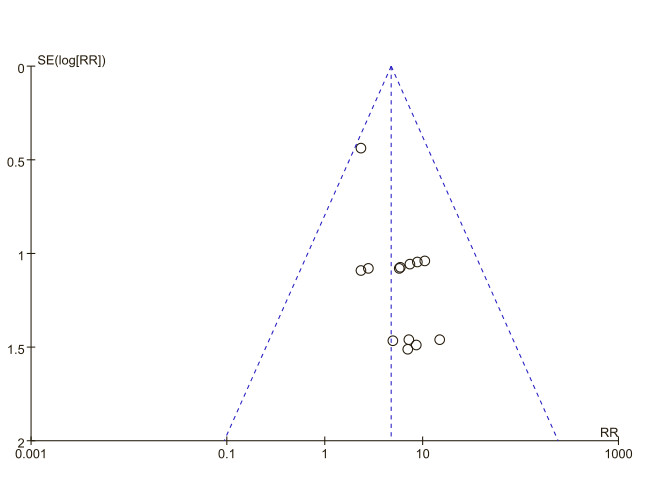
**

**Figure S6.** **The funnel plots for relative risk of high grade pneumonitis**

**
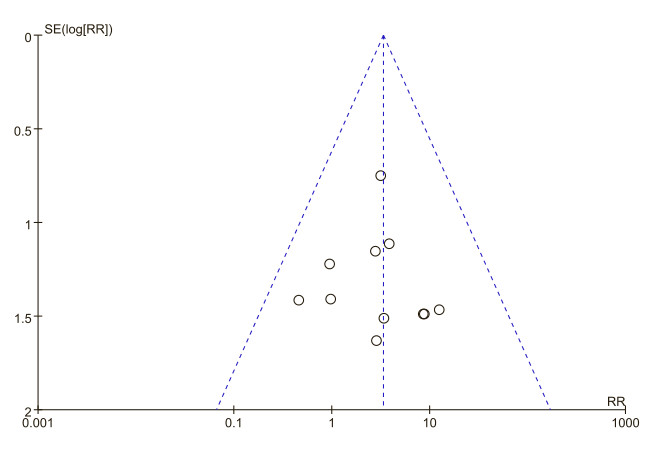
**
